# Hsa_circ_0001445 works as a cancer suppressor via miR‐576‐5p/SFRP1 axis regulation in ovarian cancer

**DOI:** 10.1002/cam4.5317

**Published:** 2022-10-19

**Authors:** Yuhong Wu, Jinhua Zhou, Yan Li, Xiu Shi, Fangrong Shen, Mingwei Chen, Youguo Chen, Juan Wang

**Affiliations:** ^1^ Department of Obstetrics and Gynecology The First Affiliated Hospital of Soochow University Suzhou China; ^2^ Clinical Research Center of Obstetrics and Gynecology Jiangsu Key Laboratory of Clinical Immunology of Soochow University Suzhou China; ^3^ Jiangsu Institute of Clinical Immunology The First Affiliated Hospital of Soochow University Suzhou China; ^4^ Department of Obstetrics and Gynecology The First People's Hospital of Yancheng Suzhou China; ^5^ Department of Urology The First Affiliated Hospital of Anhui Medical University Hefei China

**Keywords:** ceRNA, hsa_circ_0001445, metastasis, miR‐576‐5p, ovarian cancer, SFRP1

## Abstract

**Background:**

Ovarian cancer (OC) has high mortality and morbidity. Circular RNA (circRNA) can deeply impact the tumor occurrence and growth. The pathogenic activity of one particular circRNA, *hsa_circ_0001445 (hcR1445)*, in OC remains unclear and was therefore analyzed in this study.

**Methods:**

Human OC tissue specimens and cell lines (SKOV3, HO8910, and OVCAR8) were used to examine the levels of *hcR1445* and the microRNA *miR‐576‐5p* using polymerase chain reaction. The 5‐ethynyl‐2′‐deoxyuridine, flow cytometry, cellular scratch test, CCK‐8, and Transwell migration assays were used to examine the biological activities of *hcR1445* and *miR‐576‐5p* on cell apoptosis, invasion, migration, and proliferation in OC cells. Protein expression of WNT/β‐catenin and secreted frizzled‐related protein 1 (SFRP1) were tested using Western blot analysis. The potential interactions of *miR‐576‐5p/SFRP1* and *hcR1445/miR‐576‐5p* were evaluated using a dual‐luciferase report assay. The effect of *hcR1445* on OC growth and metastasis was further determined using an OC tumor xenograft model in vivo.

**Results:**

*hcR1445* level was declined in OC cells and tissues. *hcR1445* reduced cellular invasion, proliferation, and migration by blocking the ability of *miR‐576‐5p* to upregulate SFRP1 expression and consequently prohibit WNT/β‐catenin signal transduction. *hcR1445* upregulation suppressed OC growth, development, and intraperitoneal metastasis in vivo.

**Conclusion:**

*hcR1445* acts an antioncogene by targeting the *miR‐576‐5p*/SFRP1 axis and blocking OC progression and development. Thus, *hcR1445* may be employed as an indicator or a possible therapeutic target in OC patients.

## Graphical abstract

*Hsa_circ_0001445* is reduced in ovarian cancer cells, resulting in an elevation of *miR‐576‐5p* that subsequently decreases SFRP1 expression. Thus, WNT/β‐catenin is triggered to improve cancer cell invasion, proliferation, and metastasis.

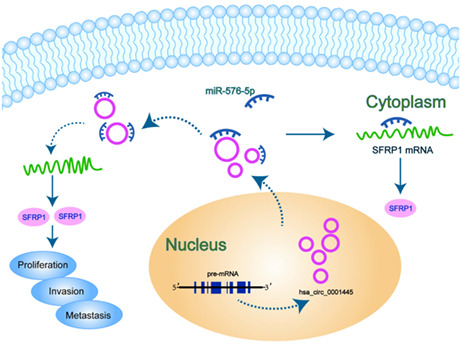

## 1 INTRODUCTION

Being a most common cancer affecting women in the world, ovarian cancer (OC) has high mortality and morbidity.^1^ Symptoms of ovary disorders are normally absent or atypical because its growth is deeply on the pelvic cavity. Most OC cases are diagnosed at advance stages with distant metastases.^2^, ^3^ Because the metastatic and recurrent potential is high in OC cases,^4^ the survival rate of recurrent OC is low and prognosis is poor. Thus, understanding the underlying etiology of the progression and development in OC is crucial for early diagnosis and effective therapy.^4^ Although many studies have explored possible mechanisms of OC,^5^, ^6^ the precise mechanism remains unclear.

*Circular RNA* (*circRNA*) belongs to a *nonexpression code RNA* (*ncRNA*) structured with an endogenous closed covalent loop. Being different to linear *RNAs*, circRNAs do not have either a 5′cap or 3′ poly‐A tail, are more stable, and cannot be easily degraded by RNA exonuclide.^7^ Although the bioactivities of *circRNAs* remain to be fully defined, some articles have revealed that *circRNAs* involve in many cellular actions, such as transcriptional regulation, molecular metabolism, RNA modification, and protein translation.^8^, ^9^ *CircRNAs* can act as *microRNA* (*miRNA*) sponges because *circRNAs* are rich in *miRNA* link sites.^10^ *CircRNAs* can competitively associate to *miRNA*‐binding sites on mRNA to increase target gene expression, which is defined as *competitive endogenous RNA* (*ceRNA*).^11^ A well‐known example is *ciRS‐7*. With over 70 constant link sites in *miR‐7*, *ciRS‐7* is generously produced in the mammalian brain. In human cell lines, decreased expression of *CDR1as* results in a decrease of *mRNAs* containing *miR‐7* link sites, indicating that *CDR1as* acts as a sponge of *ceRNA* for *miR‐7*.^10^, ^12^ Thus, through interaction with disease‐related *miRNAs*, *circRNAs* act essential regulatory works in malignancies.

*Hsa_circ_0001445* (also known as *circSMARCA5,* ^13^ *hcR1445*) originated from the *SMARCA5* gene is on the *chr4: 144*464662–*144,465,125*. According to previous studies, *hcR1445* was revealed to be declined in various malignant tumors.^13^, ^14^, ^15^ As a tumor‐inhibiting gene, *hcR1445* has been shown to substantially block cellular invasion, migration, and proliferation of tumor cells, serving as a sponge for certain *miRNAs*.^16^ However, the underline mechanism of the *hcR1445* in OC remains unclear. Here, we explored the pathological activity of *hcR1445* and provide new insights to the possible mechanisms by which *hcR1445* plays a role in OC.

## 2 MATERIALS AND METHODS

### 2.1 Subjects

Inpatients who were diagnosed with OC and who underwent oophorectomy in our hospital from July 2019 to June 2021 were enrolled in this study. Patient general information and clinical pathological data were recorded. Patients who had other cancers or therapeutic history of other cancers were excluded from this study. Patients who had other benign tumors with normal ovarian pathological tests and who underwent adnexectomy were included as controls. All sectioned ovary tissue specimens were collected and frozen with liquid nitrogen and kept in an −80°C freezer. All investigated patients provided signed written informed consents. The project was authorized by the Ethics Committee of The First Affiliated Hospital of Soochow University (No. 2021027).

### 2.2 Cell culture

Human OC cell lines, HO8910, SKOV3, and OVCAR8, were provided by the Institute of Cell Biology of The Chinese Academy of Sciences. The normal human ovarian surface epithelial cell line IOSE80 was obtained from GAINING. The cells were cultivated in complete RPMI 1640 culture medium containing 10% fatal bovine serum (FBS) (VivaCell), 100 μgg/ml streptomycin sulfate, and 100 U/ml penicillin and grown at 37°C and 5% CO_2_.

### 2.3 Small‐interfering (si)RNA

The overexpression plasmid *pLVX‐hcR1445* was synthesized by Fubio Biological Technology. Lentiviral constructs were co‐transfected with *pMD2G* and *psPAX2* into 293 T cells (ATCC) using Lipofectamine® 3000 (ThermoFisher). The virus particle‐containing supernatant was then harvested 48 h late after transfection. Subsequently, the infectious lentivirus was transfected into the SKOV3 or the HO8910 cells. Stable transfected cells were achieved through puromycin (4 μg/ml) (Invitrogen) selection for 2 weeks. SiRNA control (*si‐NC*), si‐RNAs (including *si‐hcR1445* and *si‐SFRP1*), and *miR‐576‐5p* mimics and inhibitor were constructed by Gene Pharma. The target *siRNAs*, *miRNA* inhibitors, or mimics and plasmids were co‐transfected by Lipofectamine® 3000 based on kit's instructions. The chemically synthesized targeting gene sequences were *pLVX‐hcR1445 sense: 5’‐CGCAAATGGGCGGTAGGCGTG‐3′, si‐hcR1445#1 sense*: *5′‐ACAAAAGGGAGGCUUGUGGTT‐3′, si‐hcR1445#2 sense*: *AAAACAAAAGGGAGGCUUGTT, si‐SFRP1 sense*: *GCUACAAGAAGAUGGUGCUTT*, respectively.

### 2.4 Reverse transcription‐quantitative polymerase chain reaction (PCR)

Total RNAs were isolated from the cancer samples and OC cells with Trizol regent (Invitrogen). One μg of total RNA underwent reverse transcription (RT) using the Prime Script RT Reagent Kit (Takara). *hcR1445* levels were evaluated by the SYBR Premix Ex Taq II polymerase chain reactionPCR kit (Takara) with a PCR detection instrument (CFX96 Real‐Time from Bio‐Rad) analyzed by the 2‐DDcT2 method.^17^ Corresponding primers are shown in Table S1.

### 2.5 Cellular proliferation assay

CCK‐8 was employed to assay the cellular proliferation. Briefly, cells to be detected were stained with the CCK‐8 Regent (New Cell & Molecular Biotech) for 1 h in a 96‐well culture plate. OD 450 was evaluated in a microplate reader.

Cellular proliferation was also tested using the 5‐ethynyl‐2′‐deoxyuridine (EdU) labeling assay. Briefly, cells to be detected were reacted with 500 μEdU regent (50 μM) for 2 h in an incubator at 37°C with humidified 5% CO_2_ atmosphere, and then fixed with para formaldehyde buffer (4%) for 15 min, incubated in 0.2% Triton X‐100 for 0.5 h, incubated with 200 μl Click reaction solution for 0.5 h in a dark environment, reacted with Hoechst solution for 10 min, and visualized under a fluorescence microscope.

### 2.6 Cell migration test

Cells to be detected were synchronized with basal medium once the cells reached 100% confluence. A straight wound line was gently scratched by a pipette tip. Pictures of the cells around the wound were taken at 0 and 24 h. The migrated cells in the wound area were calculated using *ImageJ* software.

### 2.7 Transwell assay

Cellular invasion was assayed using a Transwell. Cells were seeded in the upper chamber and cultured with 200 μl RPMI 1640 base medium combined with or without matrigel matrix (Corning). 20% FBS containing RPMI 1640 medium was dropped into the lower chamber. Twenty four hours late after incubation, the cells that migrated toward the lower chamber through the Transwell membrane were fixed with 4% paraformaldehyde for 15 min, stained with Violet Regent, and analyzed under an optical microscope.

### 2.8 Flow cytometry assay

The *siRNAs‐* or *plasmids‐*transfected cells were collected 72 h after transfection, rinsed with phosphate buffered saline (PBS), and mixed with 1 × binding buffer (5 μl Annexin V‐FITC and 5 μl propidium iodide from BD 556547 apoptosis assay kit [BD]) for 15 min under a light‐avoiding conditions. The cells were incubated in 400 μl Annexin V‐FITC. The fluorescence was read using the CytoFLEX S flow cytometer (Beckman).

### 2.9 Immunoblot examine

Cells to be detected were homogenized with radio‐immunoprecipitation assay buffer (Sigma‐Adrich). Each 20 μg protein sample was electrophoresed and transferred onto poly‐vinylidene fluoride membranes. Following, the membranes were blocked in 5% bovine serum albumin (BSA) buffer for 1 h, reacted with primary antibodies overnight at 4°C. In the next day, the membranes were triplicate rinsed with 1 × Tris‐buffered saline with 20% Tween (TBS‐T) buffer (Sigma‐Adrich) and then reacted with the corresponding second antibodies for another hour and again triplicate rinsed with TBS‐T buffer. Finally, the membranes were stained with an enhanced chemiluminescence reagent (Yeasen Biotechnology) to visualize the target protein bands.

### 2.10 Dual‐luciferase reporter assay

Either the wild type (WT) or the mutant type (MUT) of binding sequences of the *hcR1445* or *SFRP1* were ligated into the *PmirGLO* by following the protocol of Dual‐Luciferase Reporter Assay System (Promega). After co‐transfection of the MUT or WT *hcR1445* or *SFRP1* plasmids with *miR‐576‐5p* mimics or miR‐scramble (*miR‐NC*) for 2 days, the cells were homogenized with the Lysis Buffer (in the kit). Luciferase activity was then examined in a microplate reader.

### 2.11 RNA immunoprecipitation assay

RNA immunoprecipitation (RIP) assay was applied to detect the association between *hcR1445* and *miR‐576‐5p* or *miR‐576‐5p* and *SFRP1* (EZ‐Magna RIP RNA‐Binding Protein Immunoprecipitation Kit form Millipore). HO8910 cells were homogenized in RIP lysis buffer, reacted with magnetic beads conjugated to IgG or the argonaute2 (Ago2) antibody (Millipore) at 4°C overnight. The samples were triplicate rinsed with PBS and reacted with 10% SDS and proteinase K (Sigma‐Adrich). Total RNA on the beads was extracted with TRIzol and analyzed using reverse transcription‐quantitative PCR (RT‐qPCR).

### 2.12 Animal experiments

Sixteen female BALB/c nude mice weighing 16–18 g and aged at 4–5‐weeks were randomized into the *hcR1445* overexpression and negative control groups. For subcutaneous xenograft, each five mice (group) was subcutaneously injected with 2 × 10^6^ cells (in 100 μl PBS) into the flank. Another three mice were intraperitoneally injected with 5 × 10^6^ cells (in 100 μl PBS). The size of the xenograft tumor was measured by a caliper as well as calculated with a formula: long‐diameter × short‐diameter^2^/2 every 6 days. Tumor numbers were counted, and tumor weight was recorded after 30 days The mice were then sacrificed humanely by cervical dislocation, and the grown tumors were collected for next immunohistochemistry or biochemical examines.

### 2.13 Immunohistochemistry

Tissues to be tested were immersed in 10% formalin for 24 h, embedded with paraffin, sectioned (4 μm), mounted onto glass slides, and baked for 0.5 h at 60°C. After de‐paraffinization and rehydration, the sections were blocked with 10% BSA for 2 h, reacted with 50 μl primary antibody 12 h at 4°C, rinsed three times with PBS, and reacted with 50 μl of corresponding second antibody for 1 h. After dehydration, the slides were sealed with neutral resin and analyzed under an optical light microscope. The results of the immunohistochemistry (IHC) were evaluated for both intensity and the stained area.

### 2.14 Statistical examine

SPSS 22.0 (IBM) was applied to analyze all experimental data. Clinical data are shown as percentages and analyzed with the Fisher's exact probability method and Pearson Chi‐squared test. All experimental assays were done in triplicate and repeated independently, Data are presented as M ± SD (mean ± standard deviation). The Fisher's exact probability test or *χ* ^2^ segmentation was done to compare frequencies. An unpaired Student's *t*‐test was used to estimate differences between two groups. ANOVA was used to analyze statistical values across multiple groups. All other data were analyzed using an independent‐samples *t*‐test. *p* < 0.05 was defined as significantly different.

## 3 RESULTS

### 3.1 *hcR1445* is decreased and negatively correlates with *miR‐576‐5p* in OC

**FIGURE 1:**
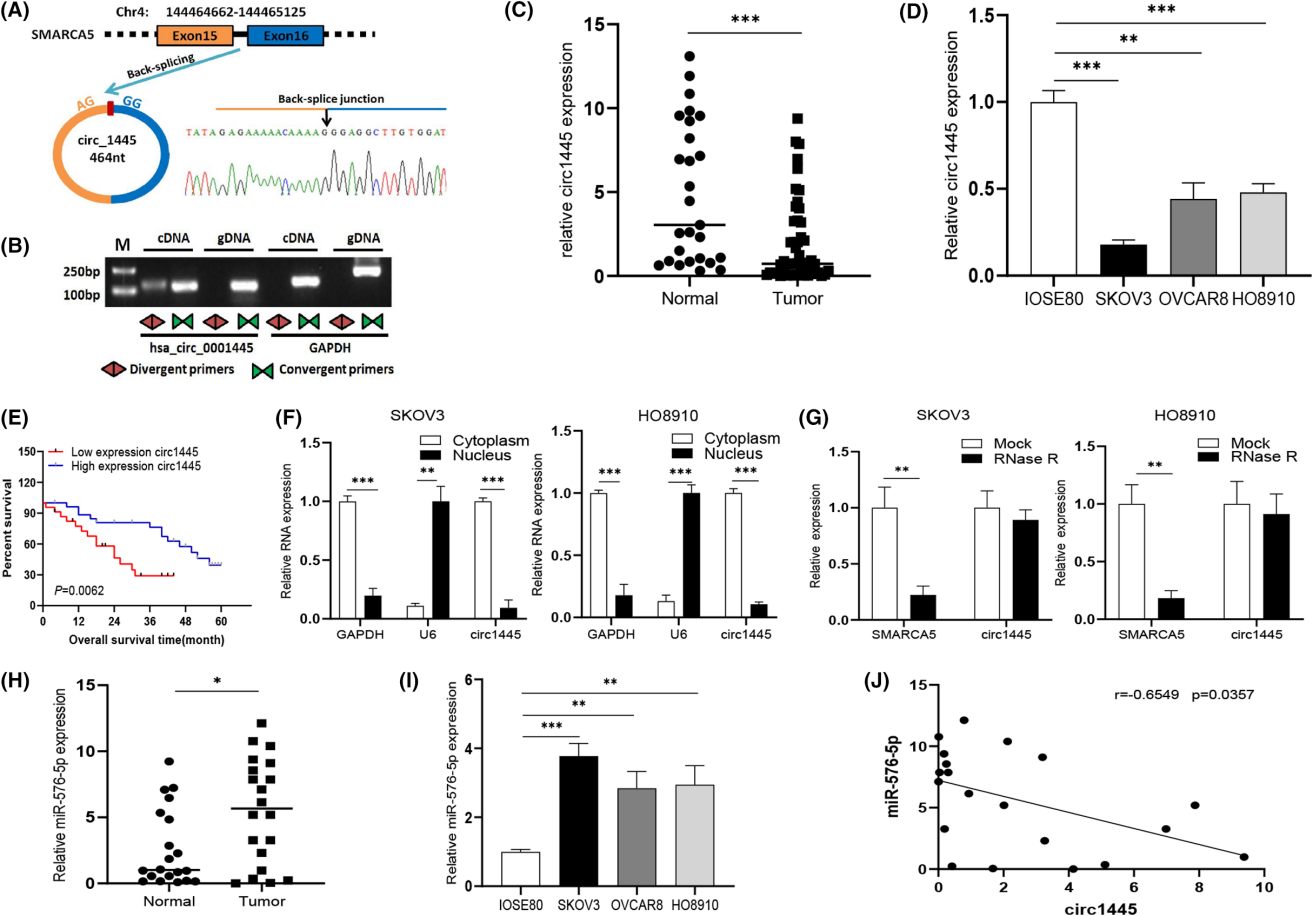
*Hsa_circ_0001445* is downregulated and *miR‐576‐5p* is elevated in ovarian cancer (OC) samples and cells. (A) Image of *hsa_circ_0001445 (circ1445)* formation via circularization of exons 15 and 16 on chromosome 4 (Chr4). Sanger sequencing analysis of the RT‐PCR products showed the splicing site of *hsa_circ_0001445*. (B) gDNA and cDNA of HO8910 cells were applied to amplify *hsa_circ_0001445* by PCR with convergent and divergent primers, separately. (C) The levels of *circ1445* were tested by RT‐qPCR in normal tissues and primary OC tissues. (D) The level of *circ1445* was examined by RT‐qPCR in IOSE80 and OC cells (SKOV3, OVCAR8, and HO8910). (E) Survival ratio of OC patients with high (blue) or low (red) levels of *circ1445*. (F) Subcellular location of *hsa_circ_0001445* was evaluated by RT‐qPCR in the nucleus and cytoplasm of SKOV3 and HO8910 cells. (G) RNA levels of *hsa_circ_0001445* and *SMARCA5* were tested by RT‐qPCR in cells treated with *RNase R*. (H, I) The expression patterns of *miR‐576‐5p* are shown in OC tissues and cells. (J) Regression analysis suggested a negative correlation of *circ1445* to the *miR‐576‐5p* (*n* = 20). *p < 0.05; **p < 0.01; ***p < 0.001.

*hcR1445* consists of 464 nucleotides from exons 15 and 16 of the human *SMARCA5* gene on *chr4:144464661–144,465,125* (Figure 1A). Sanger sequencing analysis of the RT‐qPCR products of *hcR1445* confirmed head‐to‐tail splicing with the designed size and predicted cutting site (Figure 1A). Next, cDNA or genomic DNA of the HO8910 cells was used to analyze the divergent and convergent primers for *hcR1445*. The results revealed that *hcR1445* was only detected from cDNA using the divergent primers (Figure 1B).

To examine the relative expression of *hcR1445* in OC, RT‐qPCR was performed in OC cell lines and clinical samples. Compared with normal ovarian specimens, the *hcR1445* level was notably declined in OC samples (Figure 1C), and the level of *hcR1445* was lower in OC cells comparing to IOSE80 cells (Figure 1D). We next examined the correlation of *hcR1445* and OC survival rate. Patients who had higher levels of *hcR1445* had longer survival times comparing to those who had lower *hcR1445* (Figure 1E). More patients advanced Federation International of Gynecology and Obstetrics stage patients with lymph node metastasis were found in the low *hcR1445* level group compared to the high *hcR1445* group (Table 1). Together, these data indicate that *hcR1445* production decline in the patients with OC, and low *hcR1445* production is closely associated with poor survival.

**TABLE 1:** Correlation of *hsa_circ_0001445* expression with clinicopathological parameters in ovarian cancer patients

Clinical parameters	*N* (50)	hsa_circ_0001445 expression (%)		*p*‐value
		Low	High	
Age (years)				S
≤50	23	13 (56.52)	10 (43.48)	
>50	27	15 (55.56)	12 (44.44)	
FIGO staging				0.003*
I–II	18	5 (27.78)	13 (72.22)	
III–IV	32	23 (71.88)	9 (28.12)	
Pathological type				0.045*
Serous	36	17 (47.22)	19 (52.78)	
Others	14	11 (78.57)	3 (21.43)	
Tumor size				0.474
≤5 cm	29	15 (51.72)	14 (48.28)	
>5 cm	21	13 (61.90)	8 (38.10)	
Tumor differentiation				0.063
Low	34	16 (47.06)	18 (52.94)	
Medium‐high	16	12 (75.00)	4 (25.00)	
Lymph node metastasis				0.007*
Yes	22	17 (77.27)	5 (22.73)	
No	28	11 (39.29)	17 (60.71)	

Abbreviation: FIGO, Federation International of Gynecology and Obstetrics.

* *p* < 0.05.

RNA analysis in both the nuclei and cytoplasm suggested that *hcR1445* was primarily cytosolic distribution (Figure 1F). Furthermore, the RNase R exonuclease assay indicated that linear *SMARCA5* was degraded after RNase R treatment but *hcR1445* was insensitive to RNase R treatment (Figure 1G). In contrast, the relative level of *miR‐576‐5p* was increased in the OC cells and tissues (Figure 1H,I), and the *mir‐576‐5p* level was negatively associated with the *hcR1445* level (Figure 1J).

### 3.2 *hcR1445* blocks OC cell migration, invasion, and proliferation, and promotes apoptosis

**FIGURE 2:**
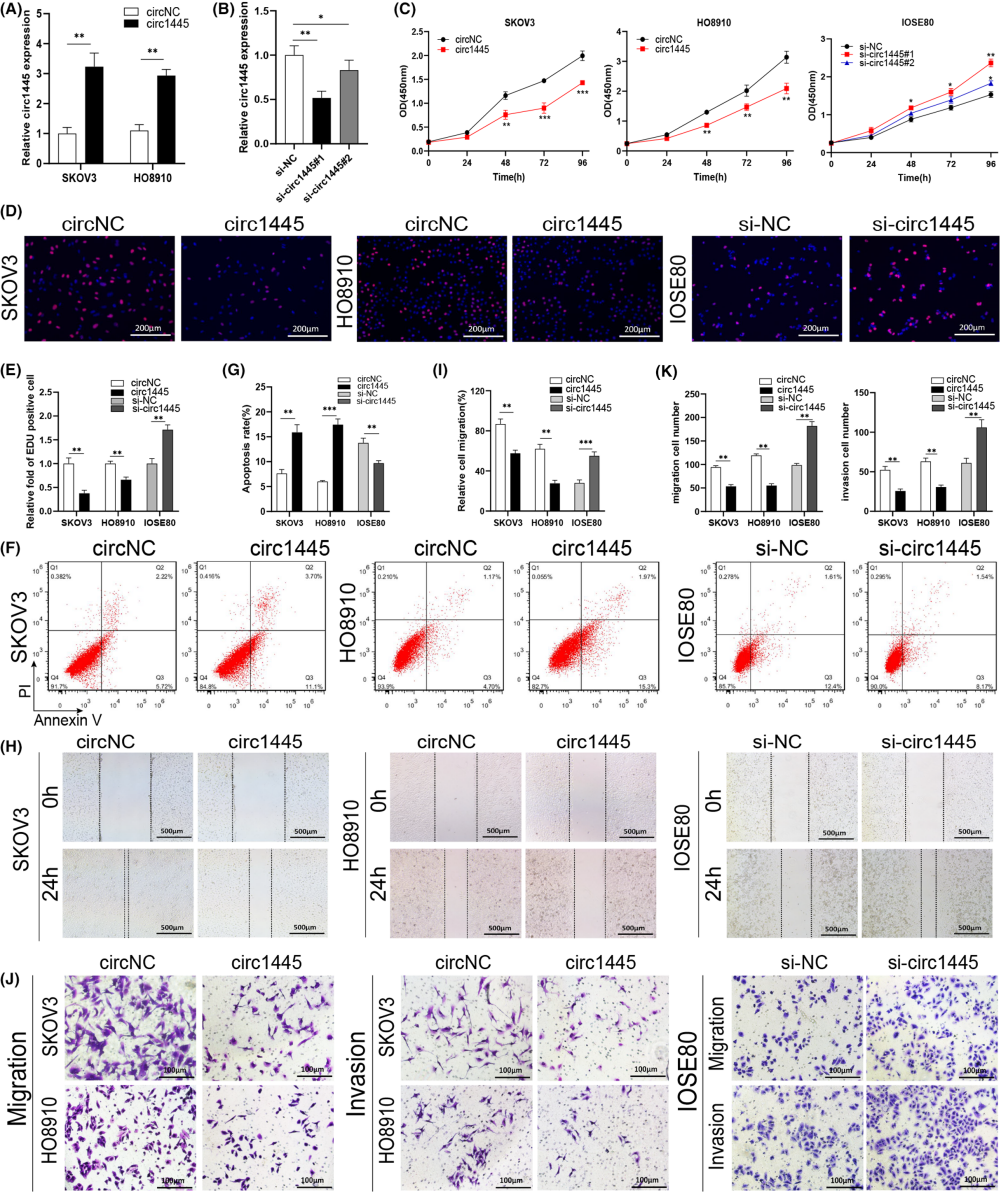
*Hsa_circ_0001445* exerts tumor‐suppressive effects in ovarian cancer (OC) cells. (A, B) The relative expression levels of *hsa_circ_0001445* were detected by RT‐qPCR in SKOV3 or HO8910 cells transfected with *circ1445* overexpression plasmids (black), *circNC* (white), *si‐circ1445‐1* (black), *si‐circ1445‐2* (dark gray), and *si‐NC* (white). (C) The proliferation of cells was examined by the CCK‐8 method in SKOV3 and HO8910 cells transfected with *circ1445* (red square), *circNC* (black circle) or the *si‐circ1445‐1‐* (red square), *si‐circ1445‐2‐* (blue triangle), and *si‐NC‐* (black circle) transfected IOSE80 cells. (D, E) Representative images showing EdU (red) analysis of cell proliferation. (F, G) Cell apoptotic level was evaluated by flow cytometry in the indicated cell lines. (H, I) Cell moving ability was examined by the wound healing test in the indicated cells. (J, K) Invasion and migration abilities of the cells were evaluated by the Transwell assay in the indicated SKOV3, HO8910, and IOSE80 cell lines. *p < 0.05; **p < 0.01; ***p < 0.001.

RT‐qPCR analysis showed that the level of *hcR1445* was significantly increased in the *hcR1445* stably overexpressed SKOV3 and HO8910 cells (Figure 2A), however, the levels significantly decreased after transfection of *si‐hcR1445* in the normal human IOSE80 ovarian epithelial cells (Figure 2B).

The CCK‐8 detection suggested that *hcR1445* significantly inhibited cellular proliferation in SKOV3 and HO8910 cell lines. However, downregulation of *hcR1445* (with either siRNA construct used) significantly enhanced cell proliferation after 0–96 h transfection in the IOSE80 cells (Figure 2C). Since siRNA #1‐mediated knockdown of *hcR1445* #1 was more efficient than the si‐RNA #2, the #1 construct was selected for subsequent experiments. Cellular proliferation was also examined using the EdU assay after 72 h transfection. Compared with controls, the EdU‐positive cell were declined in the *hcR1445*‐expressed SKOV3 and HO8910 cells, but increased in the *si‐hcR1445* transfected IOSE80 cells (Figure 2D,E). Flow cytometry detection was applied to measure cellular apoptosis. Consistently, the results revealed that *hcR1445* greatly enhanced the relative apoptosis ratios in SKOV3 and HO8910 cell lines. However, knockdown of *hcR1445* restrained apoptosis in the IOSE80 cell line (Figure 2F,G).

The scratch‐wound and Transwell assays were employed to detect the metastatic capacity of the OC cell lines. The scratch‐wound assay revealed that cellular migration was tremendously declined in the *hcR1445* expression group compared to the *circNC* group. Knockdown of *hcR1445* increased the cell migration ratio (Figure 2H,I). Additionally, Transwell assays revealed that neither migration nor invasion were reduced in the *hcR1445* group, but increased in the *si‐hcR1445* group (Figure 2J,K).

### 3.3 MiR‐576‐5p induces OC cell migration, proliferation, invasion, and blocks apoptosis

**FIGURE 3:**
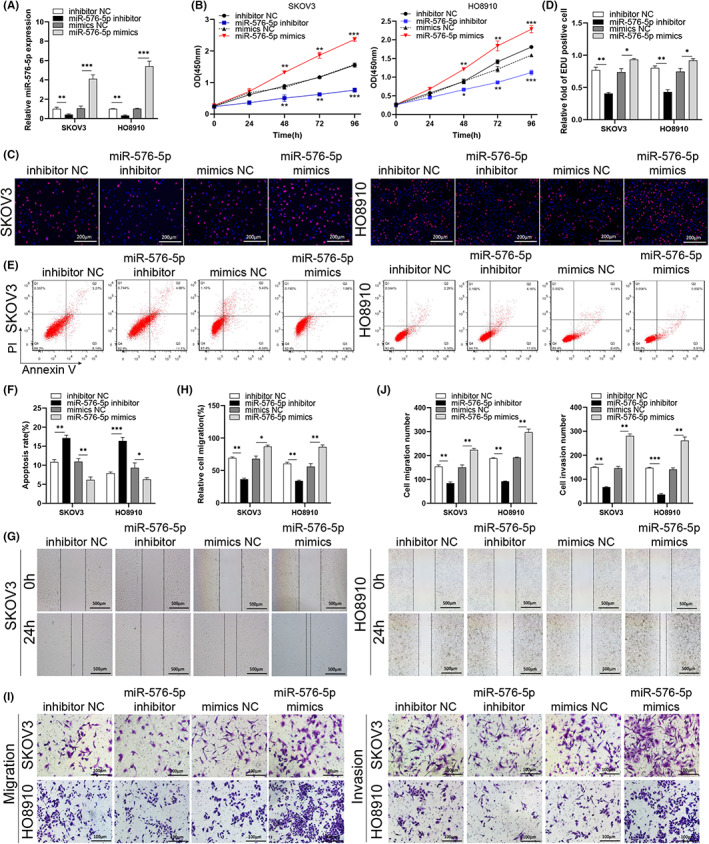
Knocking‐down or overexpression of miR‐576‐5p suppresses or promotes cell progression, respectively. (A) The level of *miR‐576‐5p* was detected by RT‐qPCR in the indicated cells. (B) Cell proliferation was detected using the CCK‐8 assay in the indicated ovarian cancer (OC) cells. (C, D) Proliferation of the cells was evaluated with EdU labeling (red) in the indicated cells. (E, F) Flow cytometry was used to detect the percentage of the *miR‐576‐5p* inhibitor‐ or mimics‐transfected apoptotic OC cells. (G–J) Cellular moving ability and invasive ability were evaluated using the wound healing (G, H) and Transwell (I, J) assays in the *miR‐576‐5p* inhibitor or mimics transfected SKOV3 and HO8910 cell lines. *p < 0.05; **p < 0.01; ***p < 0.001.

Next, we evaluated *miR‐576‐5p* activity in OC. As expected, RT‐qPCR assays revealed that the relative production of *miR‐576‐5p* markedly increased or decreased after miR‐576‐5p inhibitor or mimic transfection, respectively (Figure 3A). The CCK‐8 assay demonstrated that *miR‐576‐5p* mimics accelerated cell proliferation in both OC cells, while blocking *miR‐576‐5p* with the inhibitor restrained OC cell proliferation during 0–96 h after transfection (Figure 3B). Consistently, more EdU positive cells were found in the *miR‐576‐5p* mimics group, and fewer EdU‐labled cells were observed in the *miR‐576‐5p* inhibitor group (Figure 3C,D). We also found that increasing *miR‐576‐5p* levels blocked OC cell apoptosis while reducing *miR‐576‐5p* levels accelerated OC cell apoptosis (Figure 3E,F).

Cellular migration assay suggested that *miR‐576‐5p* mimics obviously increased cell migration in the two OC cell lines. *MiR‐576‐5p* knockdown decreased cell migration comparing to the normal group (Figure 3G,H). The Transwell analysis suggested that either cell migration or invasion was remarkably elevated in the *miR‐576‐5p* mimics transfected group, but was suppressed in the *miRNA‐576‐5p* inhibitor group comparing to the miRNA transfected control group (Figure 3I,J). These data indicate that higher *miR‐576‐5p* levels accelerate tumor metastasis, and reducing *miR‐576‐5p* levels can block OC cell metastasis.

### 3.4 *hcR1445* directly binds with *miR‐576‐5p* to prohibit OC cells progression

**FIGURE 4:**
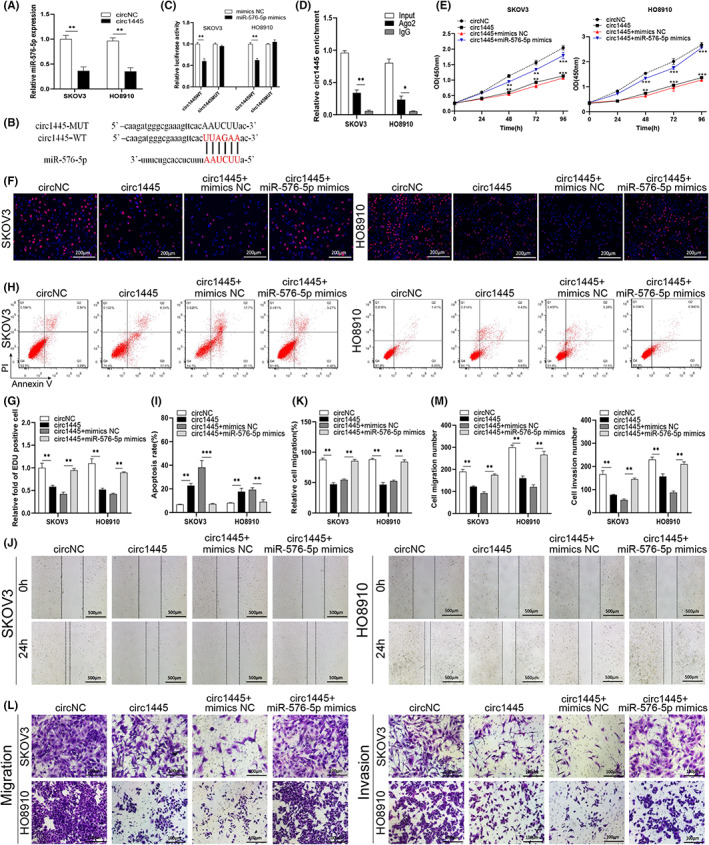
*Hsa_circ_0001445* regulates cell proliferation, apoptosis, invasion, and migration of ovarian cancer (OC) cells by interacting with *miR‐576‐5p*. (A) The expression of the *miR‐576‐5p* was examined by RT‐qPCR in the SKOV3 or HO8910 cells transfected with *circ1445* (black) or *circNC* (white). (B) Bioinformatics predicted the association sites between *miR‐576‐5p* and *hsa_circ_0001445*. (C) Binding of *miR‐576‐5p* and *hsa_circ_0001445* was examined by dual‐luciferase tests in the SKOV3 and HO8910 cells co‐transfected with *circ1445‐Wt* or *circ1445‐Mut* and *miR‐576‐5p* mimics or NC, respectively. (D) Interaction of *hsa_circ_0001445* and Ago2 was detected by immunoprecipitation in the Ago2‐pull down lysate from the OC cells. (E–G) The proliferation of the cells was estimated with the CCK‐8 kit (E) and EdU labeling (F, G) in the SKOV3 and HO8910 cells co‐transfected with *circ1445+ miR‐576* mimics or inhibitor. (H, I) The apoptosis rate was estimated using flow cytometry in the *circ144*5 and *miR‐576* inhibitor or mimics co‐transfected SKOV3 and HO8910 cells. (J–M) Cell migration and invasion were examined using the wound healing (J, M) and Transwell (L, M) assays in the indicated SKOV3 and HO8910 cells. *p < 0.05; **p < 0.01; ***p < 0.001.

CircRNAs are reported to work as a sponge for miRNA activity. Thus, we next analyzed the correlation of *hcR1445* and *miR‐576‐5p*. Interestingly, *hcR1445* expression resulted in a lower level of *miR‐576‐5p* comparing to the empty vector‐transfected cells (Figure 4A), indicating that *hcR1445* is negatively associated with *miR‐576‐5p*. According to the bioinformatics databases, we found that *hcR1445* possesses some complementary binding sites to *miR‐576‐5p* (Figure 4B). Dual‐luciferase assays showed interactions between *hcR1445* and *miR‐576‐5p* and that *miR‐576‐5p* was suppressed compared to *hcR1445‐WT* but not *hcR1445‐MUT* (Figure 4C). Furthermore, *hcR1445* was detected in the Ago2 antibody immunoprecipitate, implying that *hcR1445* is associated with miRNAs via Ago2 (Figure 4D). No obvious difference was found between co‐transfections of mimics NC with *hcR1445‐WT* or *hcR1445‐MUT*. Summarily, these results demonstrate that *hcR1445* directly associates with *miR‐576‐5p*.

We next explored the effects of the *hcR1445* and *miR‐576‐5p* interaction on OC cells. The CCK‐8 examine showed that *hcR1445* obviously reduced the OC cell proliferation. Co‐transfection of *hcR1445* with *miR‐576‐5p* mimics also suppressed OC cell proliferation, but significantly reversed the blocked effect to some extent (Figure 4E). *hcR1445* also decreased the number of EdU‐labled cells comparing to the *circNC* group. The *hcR1445 + miR‐576‐5p* mimics group had more EdU‐labled cells than the *hcR1445 + miR‐NC* group, which suggests that *miR‐576‐5p* mimics rescued the inhibitory effect of *hcR1445* on OC cell progression (Figure 4F,G). In addition, compared with the *circNC* group, cellular apoptosis in the *hcR1445* overexpression group was significantly accelerated. Co‐overexpression of *hcR1445* and *miR‐576‐5p* mimics partially declined the percentage of apoptosis induced by *hcR1445* (Figure 4H,I).

The scratch‐wound assays demonstrated that overexpression of *hcR1445* reduced the relative cell mobility compared with the *circNC* group. The *hcR1445*‐induced decrease in cell migration was partially restored by co‐expression of the *miR‐576‐5p* mimics in the OC cells (Figure 4J,K). Similarly, the Transwell test suggested that *hcR1445* significantly decreased cell invasion and migration compared with the *circNC*. *The miR‐576‐5p* mimics partially reversed the *hcR1445‐*induced OC cell invasion and migration (Figure 4L,M).

Collectively, our data suggest that *hcR1445* inhibits cellular migration, invasion, and proliferation, as well as promotes cell apoptosis in OC cells by targeting *miR‐576‐5p*, which could be rescued by *miR‐576‐5p* mimics.

### 3.5 *hcR1445* mediates OC cell progression on sponging *miR‐576‐5p* and regulates SFRP1 and WNT/β‐catenin pathway

**FIGURE 5:**
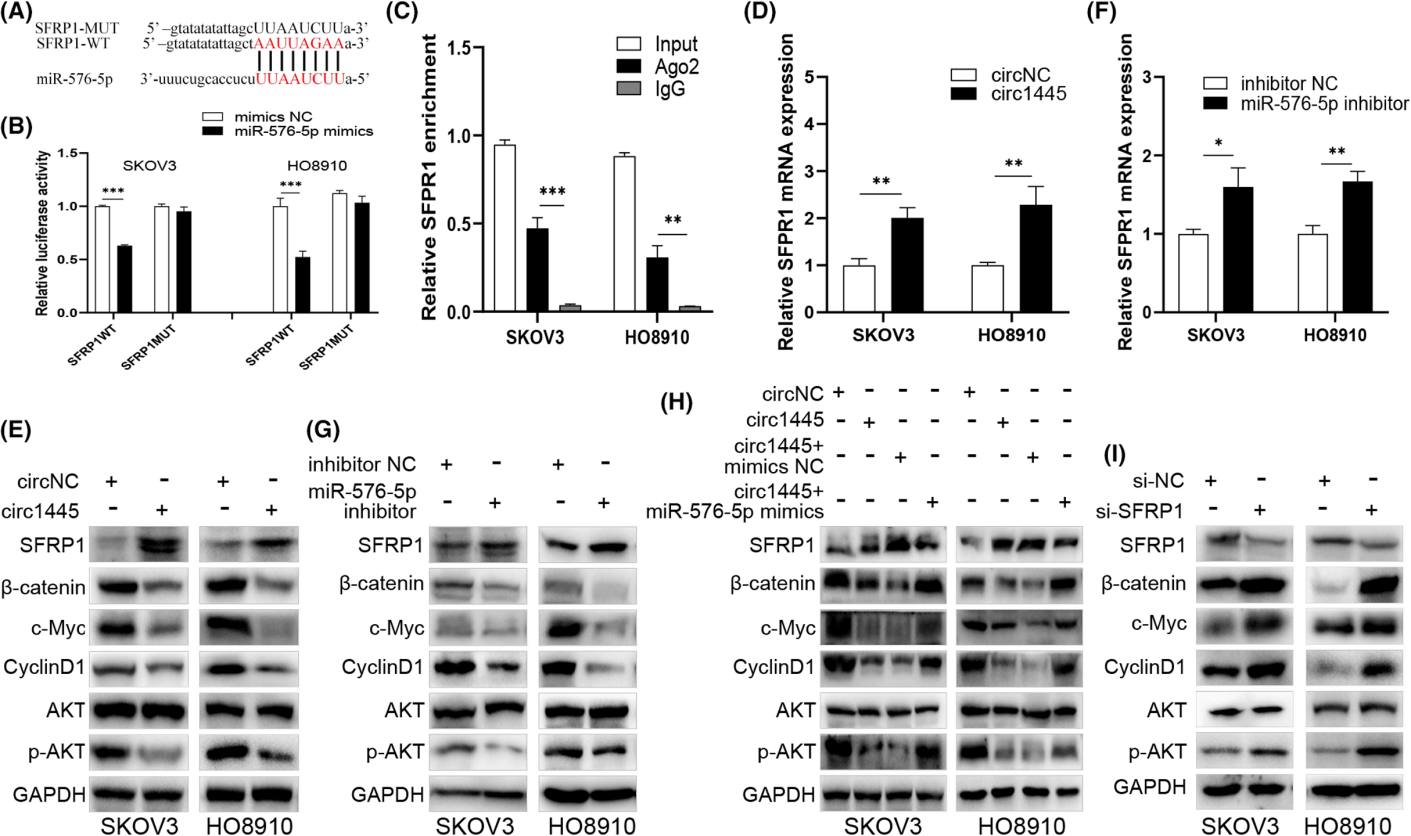
*Hsa_circ_0001445* regulates SFRP1 production and the signaling of WNT/β‐catenin via sponging *miR‐576‐5p*. (A) Bioinformatics analysis was applied to predict the binding sites of the *SFRP1* mRNA to the *miR‐576‐5p*. (B) Interaction of *SFRP1* mRNA and *miR‐576‐5p* was estimated by dual‐luciferase tests in the ovarian cancer (OC) cells co‐transfected with *SFRP1‐Wt* or *SFRP1‐Mut* and *miR‐576‐5p* mimics or *NC*. (C) Interaction of SFRP1 and Ago2 was detected by immunoprecipitation. The SFRP1 level was examined in the Ago2 antibody‐immunoprecipitate from OC cells. (D, E) The expression level of *SFRP1* mRNA (D) and protein (E) was analyzed by RT‐qPCR (D) and Western blot (E), respectively, in the *hsa_circ_0001445* transfected cells. The CyclinD1, p‐AKT, AKT, β‐catenin, and c‐Myc levels were evaluated with Western blot. (F, G) The mRNA production of *SFRP1* (F) and protein expression of SFRP1, AKT, p‐AKT, c‐Myc, β‐catenin, and CyclinD1 (G) were analyzed in the *miR‐576‐5p* inhibitor‐transfected cells transfected. (H) Interference of *circ1445* or *miR‐576‐5p* on SFRP1, AKT, p‐AKT, CyclinD1, β‐catenin, and c‐Myc proteins were also detected by Western blot. (I) Transfection efficiency of si‐SFRP1 on AKT, p‐AKT, β‐catenin, c‐Myc, and CyclinD1 was, respectively, evaluated by Western blot in the OC cells. *p < 0.05, **p < 0.01, ***p < 0.001.

Bioinformatics analysis predicted some potential mutual link sites between SFRP1 and *miR‐576‐5p* (Figure 5A). The relative luciferase activity of the SFRP1‐WT and the SFRP1‐MUT treated with *miR‐576‐5p* mimics and the negative control confirmed that *miR‐576‐5p* mimics treatment significantly reduced the luciferase activity of the SFRP1‐WT but not the SFRP1‐MUT (Figure 5B). Moreover, SFRP1 was also enhanced in the Ago2 antibody‐immunoprecipitate comparing to the IgG‐immunoprecipitate, indicating that SFRP1 could bind to *miRNAs* via Ago2 (Figure 5C).

The *hcR1445‐transfected* cells had higher levels of both SFRP1mRNA and protein compared to the cells transfected with *circNC* (Figure 5D,E). SFRP1 is considered to be an antagonist of Wnt signaling.^18^, ^19^ Our experiments showed that Wnt/β‐catenin activity was related to the bioactivity of *hcR1445* in OC cells. *hcR1445* overexpression remarkably diminished some pro‐oncogenic proteins, including β‐catenin, p‐AKT, c‐Myc, and CyclinD1 (Figure 5E). *The miR‐576‐5p* inhibitor significantly promoted SFRP1 expression in OC cells (Figure 5F,G). In addition, overexpression of *hcR1445* significantly promoted SFRP1 expression, and the *miR‐576‐5p* mimics restored the *hsa_circ_0001445*‐induced SFRP1 expression to a certain extent (Figure 5H), indicating that *hsa_circ_0001445* can regulate SFRP1 expression *by* sponging *miR‐576‐5p* in OC cells. We also found that knockdown of SFRP1 activated Wnt/β‐catenin signal transduction in the OC cells (Figure 5I).

**FIGURE 6:**
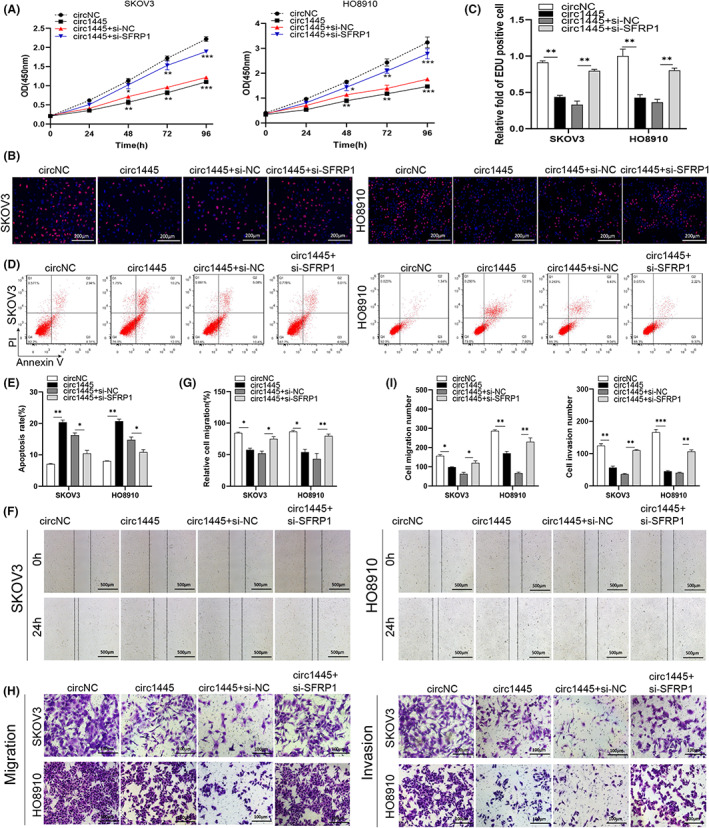
Knockdown of SFRP1 inhibited cancer cell migration, proliferation, and invasion induced by *hsa_circ_0001445*. (A–C) The CCK‐8 (A) and EdU (B, C) assays were used to estimate cell proliferation in cells overexpressing *hsa_circ_0001445* (*circ1445*). (D, E) The cellular apoptotic ability was assayed by flow cytometry in the *si‐SFRP1* and *hsa_circ_0001445* treated ovarian cancer (OC) cells. (F–I) The migrative ability (F, G) and invasive ability (H, I) were tested using both the wound healing and Transwell tests in the *hsa_circ_000144*5‐ or *si‐SFRP1‐treated OC cells*. *p < 0.05, **p < 0.01, ***p < 0.001.

Interestingly, co‐transfection with si‐SFRP1 significantly rescued the *hcR1445*‐mediated block of cellular proliferation (Figure 6A,B,C), migration (Figure 6F,G), and invasion (Figure 6H,I) in the OC cells. SFRP1 knockdown also reversed the *hcR1445*‐suppressed apoptosis in the OC cells (Figure 6D,E). Collectively, these results elucidate how *hcR1445* restrains malignant phenotypes in OC cells by regulating SFRP1.

### 3.6 Overexpression of *hcR1445* inhibits OC cell tumorigenicity and intraperitoneal metastasis in vivo

**FIGURE 7:**
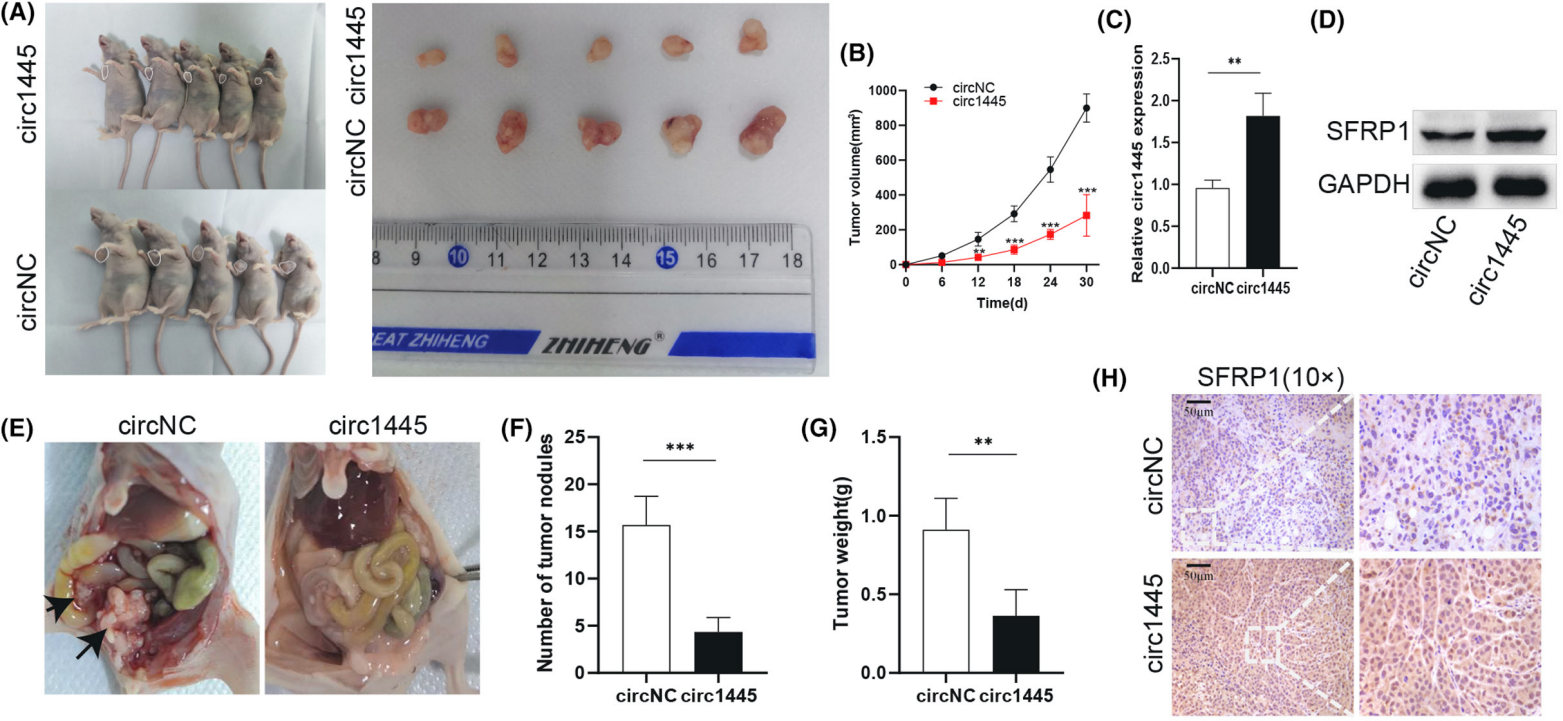
*Hsa_circ_0001445* inhibits ovarian cancer (OC) tumor growth and metastasis in vivo. (A) Images of the mice (left panels) and the isolated tumors (right panel) are shown for the mice that were subcutaneously implanted with either *circ1455*‐ (left‐up panel) or *circNC*‐ (left‐down panel) transfected OC cells. (B) The image shows the growth curves of the *circ1455*‐tumors (red square) and the *circNC*‐tumors (blue circle). (C) RT‐qPCR results show the levels of the *circ1445*‐tumor (black) and the *circNC*‐tumor (white). (D) Representative results of Western blot for SFRP1 protein expression in the indicated samples. (E) Representative images show intraperitoneal metastases in the c*irc1455* tumor (right) or *circNC* tumor (left) mice. (F) The number of intraperitoneal metastatic tumor nodules in the *circ1445*‐tumor (black) and the *circNC*‐tumor (white) mice. (G) Tumor weight of the intraperitoneal metastatic tumor nodules is quantified in the *circ1445*‐tumor (black) and the *circNC*‐tumor (white) mice. (H) IHC analysis of SFRP1 expression in the *circ1445*‐tumor (low panels) and the *circNC*‐tumor (up panels). *p < 0.05, **p < 0.01, ***p < 0.001.

**FIGURE 8:**
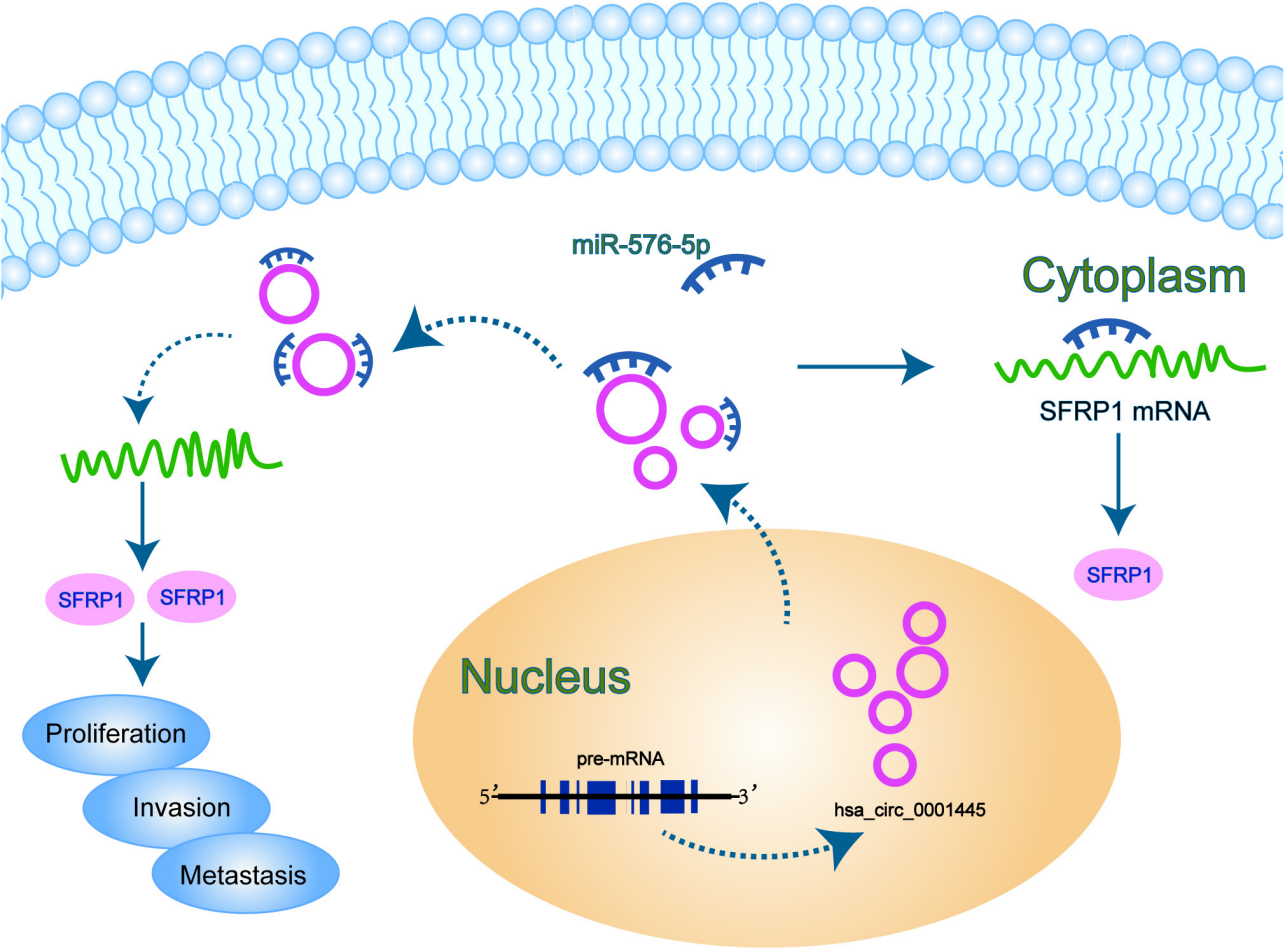
Schematic model of the role of *hsa_circ_0001445*/*miR‐576‐5p*/SFRP1 axis in ovarian cancer (OC). *Hsa_circ_0001445* is reduced in OC cells, resulting in an elevation of *miR‐576‐5p* that subsequently decreases SFRP1 expression. Thus, WNT/β‐catenin is triggered to improve cancer cell invasion, proliferation, and metastasis.

To confirm whether *hcR1445* regulates tumorigenicity and intraperitoneal metastasis of OC cells in vivo, intraperitoneal and subcutaneous xenografts were established in female nude mice using HO8910‐*circ1445* cells (high *hcR1445* expressing cell strain) and HO8910‐*circNC* cells. The results showed that cancer growing was obviously slow down in the *circ1445* groups than in the *circNC* groups (Figure 7A,B). In addition, overexpression of *hcR1445* reduced the number of tumor nodes and intraperitoneal tumor weight compared to the HO8910‐*circNC* group (Figure 7C,D,E). Further, Western blot and RT‐qPCR analyses confirmed that over‐expression of *hcR1445* significantly up‐regulated SFRP1 expression in the OC cells in vivo (Figure 7F,G). In addition, IHC analysis suggested that the relative expression of SFRP1 was elevated in the *circ1445*‐transfected tumors but was reduced in the *circNC*‐transfected tumors (Figure 7H). In summary, overexpression of *hcR1445* could inhibit OC growth and OC metastasis in vivo, and *hcR1445* works as a cancer suppressor through regulating the *miR‐576‐5p*/SFRP1 axis in OC (Figure 8).

## 4 DISCUSSION

OC is usually detected at an advanced stage as a result of distant metastasis because there are no early symptoms and due to a lack of early detection biomarkers.^20^, ^21^ Due to its high morbidity and mortality, understanding the potential pathogenic mechanism of OC has great significance for improving diagnosis and therapeutic effects.^22^ Recent articles have indicated that circRNAs impact tumor pathological processes, acting as tumor drivers or tumor suppressors in different circumstances.^23^, ^24^ For instance, *circPLEKHM3* acts as an antioncogenic factor that prohibits cancer cell growth, progression, and taxol resistance by targeting *miR‐9* in OC.^25^ *Circ‐HuR* was shown to prohibit the proliferation and aggressive characteristics of gastric cancer by physically interacting with proteins,^26^ and *hsa_circ_0004214* promotes cervical tumor cell growth both in vivo and in vitro through sponging *miR‐526b*.^27^ However, the detailed mechanism of the *hcR1445 activity* in OC is completely unknown. In this study, we demonstrated that *hcR1445* levels were remarkably declined in OC cells and tissues, while *miR‐576‐5p* levels were increased. Low levels of *hsa_circ_0001445* correlated with decreased survival in OC patients. *hcR1445* blocked cell migration, invasion, and proliferation, but promoted apoptosis in OC cells via *miR‐576‐5p*/SFRP1 axis regulation. Upregulation of *hcR1445* inhibited the growth and abdominal metastasis of OC in vivo. Therefore, it is concluded that *hcR1445* plays a major role in the progression and development of OC.

MiRNAs have been involved in numerous cancer processes.^28^, ^29^ *MiR‐576‐5p* has been indicated to be elevated in gastric cell carcinoma and cancer and shown to function as a tumor promoter to facilitate cell migration, growth, and invasion.^30^, ^31^ Nevertheless, a detailed role of *miR‐576‐5p* has yet to be described in OC. Here, we demonstrated that *miR‐576‐5p* was elevated both in OC tissues and cells, suggesting that it may act as a cancer promoter. Further, we confirmed that *miR‐576‐5p* knockdown prohibited OC cell migration, proliferation, and invasion, but promoted apoptosis, while *miR‐576‐5p* overexpression had the opposite effects. Together, these data show that *miR‐576‐5p* plays a crucial role in OC occurrence and development. Interestingly, *miR‐576‐5p* was reported to interact with several *ncRNAs* such as *linc01133* and *linc‐PINT*.^29^, ^30^ Nevertheless, the correlation of *miR‐576‐5p* and *hcR1445* in OC remains unknown. Mechanistically, based on bioinformatics analysis, we observed that *miR‐576‐5p* was the downstream target of the *hcR1445*. The dual‐luciferase assay confirmed that *hcR1445* and *miR‐576‐5p* physically bind to each other. To further verify the potential function of the *hcR1445/miR‐576‐5p* axis in OC, *hcR1445* was overexpressed in OC cells, in which *miR‐576‐5p* expression was significantly declined. In contrast, *hcR1445* knockdown resulted in high levels of *miR‐576‐5p*, as well as the *miR‐576‐5p* inhibitor partly attenuated these effects while the *miR‐576‐5p* mimics strengthened the effects of overexpressing *hcR1445* in OC cells.

SFRP1 is part of the glycoprotein secreted frizzled‐related protein family, which is located at the *8p11.21 chromosome* region.^32^ SFRP1 is believed to be an anti‐tumor factor due to its low expression level in human cancers.^33^, ^34^ Low levels of SFRP1 are associated with transcriptional silencing by miRNAs.^35^ Actually, many studies have indicated that SFRP1 has anti‐oncogene activity and that SRFP1 extensively participates in dysregulation of cancer cell migration, proliferation, and invasion.^36^ A previous report revealed aberrant expression of SFRP1 influenced the growth and metastasis of OC.^37^ Although past researches indicated that SFRP1 was a critical antagonist of the Wnt signaling pathway,^18^, ^38^ it remains unclear if SFRP1 is an upstream regulators of the pathway in OC. In this research, we demonstrated that SFRP1 was a direct downstream target gene of *miR‐576‐5p*. The production level of SFRP1 was recovered by knockdown of *miR‐576‐5p*. Likewise, the level of SFRP1 was upregulated by overexpression of *hcR1445*. We also verified that SFRP1 mediated the function of *hcR1445*. Knockdown of SFRP1 significantly reversed the anti‐neoplastic effect of *hcR1445* to a certain extent. Based on our experimental results, we confirmed that *hcR1445* sponged *miR‐576‐5p* in a ceRNA‐dependent manner. Overexpression of *hcR1445* remarkably decreased *miR‐576‐5p* levels and subsequently increased SFRP1 expression, which further inhibited the Wnt signal transduction pathway (including β‐catenin, cyclin D1, and C‐myc). Collectively, our study provides strong evidences that *hcR1445* can act as a cancer inhibitor by miRNA sponging, which may serve as an underlying key therapeutic target in OC.

In conclusion, this study revealed that *hcR1445* is expressed at low levels in OC, which may affect OC cell migration, proliferation, invasion, and apoptosis. Furthermore, *hcR1445* may act as an important factor in the progression and metastasis of OC both in vivo and in vitro *by* targeting *miR‐576‐5p to* regulate SFRP1 and Wnt/β‐catenin signal transduction. Thus, *hsa_circ_0001445* could be further investigated as a novel and important tumor diagnostic biomarker, potentially leading to promising therapeutic approaches of OC malignancy.

## AUTHOR CONTRIBUTIONS

**Yuhong Wu:** Investigation (equal); methodology (equal); writing – original draft (equal). **Jinhua Zhou:** Data curation (equal); validation (equal). **Yan Li:** Investigation (equal); methodology (equal); resources (equal). **xiu shi:** Data curation (equal); formal analysis (equal); validation (equal). **Fangrong Shen:** Formal analysis (equal); supervision (equal). **Mingwei Chen:** Investigation (equal); methodology (equal); visualization (equal). **Youguo Chen:** Funding acquisition (equal); validation (equal); writing – review and editing (equal). **juan wang:** Methodology (equal); project administration (equal); resources (equal); supervision (equal); writing – review and editing (equal).

## FUNDING INFORMATION

The present study was funded by the Minsheng Science and Technology of Suzhou Minsheng Science and Technology (No. SYSD2019204) as well as the Project of Suzhou Science and Technology Development Plan (No. SLJ202006).

## CONFLICT OF INTEREST

No competing interests are stated by the authors.

## Supporting information


Table S1

## Data Availability

All data of the study could be obtained upon request to the communication author.
