# Supplementary material for: Hsa_circ_0001445 works as a cancer suppressor via miR‐576‐5p/SFRP1 axis regulation in ovarian cancer
Source: Cancer Med. 2022 Oct 19;12(5):5736–50. doi: 10.1002/cam4.5317 (PMC10028118; doi:10.1002/cam4.5317)
Supplement: Supplementary file 1 — Table S1 [file CAM4-12-5736-s001.docx]

**Electronic supplementary material (ESM) Table S**

ESM Table S1: The primers for real-time QPCR

| Gene | Forward or Reverse | Primer sequence |
| --- | --- | --- |

| Hsa_circ_0001445 | Forward | 5’- GATGGTCAAGCCCTACCCTG -3’ |
| --- | --- | --- |
|  | Reverse | 5’- CCCTGATGCTACTGGTTGCC -3’ |
| miR-576-5p | Forward | 5’-ACACTCCAGCTGGGATTCTAATTTCTCCAC-3’ |
|  | Reverse | 5’-CTCAACTGGTGTCGTGGAGTCGGCAATTCAGTTGAGAAAGACGT-3’ |
| SFRP1 | Forward | 5’-AGCTTGTGCTGTACCTGAAGAATGG -3’ |
|  | Reverse | 5’-CATGATGAGGAAGTGGTGGCTGAG-3’ |
| SMARCA5 | Forward | 5’-TCATTCAACCTGCTGCTCAGAAGAC -3’ |
|  | Reverse | 5’-GTGTCGGTAATCGCCAACGGATAG-3’ |
| GAPDH | Forward | 5’-CGCTCTCTGCTCCTCCTGTTC-3’ |
|  | Reverse | 5’ATCCGTTGACTCCGACCTTCAC-3’ |
| U6 | Forward | 5’-CTCGCTTCGGCAGCACA-3’ |
|  | Reverse | 5’- ACGCTTCACGAATTTGCGT-3’ |
